# Components of metabolic syndrome in patients with multiple myeloma and smoldering multiple myeloma

**DOI:** 10.1186/s12885-020-06976-1

**Published:** 2020-05-30

**Authors:** Efrat Markus, Svetlana Trestman, Yael Cohen, Yoel Angel, Yael Sofer, Moshe Mittelman, Irit Avivi, Naftali Stern, Elena Izkhakov

**Affiliations:** 1 Institute of Endocrinology, Metabolism and Hypertension, Tel Aviv Sourasky Medical Center, Tel Aviv, Israel, affiliated to the Sackler Faculty of Medicine, Tel Aviv University, Tel Aviv, Israel; 2 Institute of Hematology, Tel Aviv Sourasky Medical Center, Tel Aviv, Israel, affiliated to the Sackler Faculty of Medicine, Tel Aviv University, Tel Aviv, Israel; 3 Department of Internal Medicine C, Tel Aviv Sourasky Medical Center, Tel Aviv, Israel, affiliated to the Sackler Faculty of Medicine, Tel Aviv University, Tel Aviv, Israel; 4 Department of Internal Medicine A, Tel Aviv Sourasky Medical Center, Tel Aviv, Israel, affiliated to the Sackler Faculty of Medicine, Tel Aviv University, Tel Aviv, Israel

**Keywords:** Smoldering multiple myeloma, Hyperlipidemia, Metabolic changes

## Abstract

**Background:**

The prevalences of diabetes mellitus and hypertension, both of which are components of metabolic syndrome, are known to be increased among patients with multiple myeloma (MM), but remain undetermined among patients with smoldering MM (SMM).

**Methods:**

Changes in various components of metabolic syndrome were investigated during the follow-up of patients with either MM or SMM compared to healthy controls. The data of 153 patients (105 with MM and 48 with SMM) and 138 controls were accessed from our medical center’s records between 2008 and 2015. We analyzed the patients’ data at diagnosis (baseline) and after 1, 3, and 5 years of follow-up.

**Results:**

Patients with SMM had a significantly higher prevalence of diabetes, hypertension, and dyslipidemia at baseline compared to controls. A multivariate Cox regression analysis revealed a higher risk to develop dyslipidemia after 1, 3, and 5 years of follow-up among the SMM patients. The MM patients had a higher risk to develop diabetes after 1 year, hypertension after 5 years, and dyslipidemia after 1, 3, and 5 years of follow-up.

**Conclusions:**

These data demonstrate that patients with SMM and those with MM are more prone to develop various components of metabolic syndrome, and they stress the importance of following-up metabolic syndrome components in both groups of patients.

## Background

Multiple myeloma (MM) is a malignancy of plasma cells that comprises about 10% of hematological cancers. The incidence of MM is expected to almost double in the upcoming decades due to the aging of the population [1]. Advances in MM therapies [2] have resulted in improved survival [3, 4], thus even further increasing the prevalence of the disease. MM develops from a premalignant state called “monoclonal gammopathy of undetermined significance” from which there is a small (~ 1%) yearly chance of conversion to a malignant state [5]. Some patients (~ 14%) undergo an in-between state called “smoldering multiple myeloma” (SMM) [6, 7]. The updated definition of SMM is the presence of a serum monoclonal (M) protein of ≥3 g/dL or a urinary M protein ≥500 mg/24 h, and/or 10 to 60% clonal bone marrow plasma cells with no evidence of end-organ damage (i.e., hypercalcemia, renal failure, anemia, or lytic bone lesions and/or other myeloma-defining events) [8]. SMM has a higher risk of conversion to MM, reaching up to about 10–95% during the first 5 years after diagnosis [9], depending on SMM risk factors [10]. About one in seven persons with MM in the US had initially been diagnosed as having SMMs [7].

Since most MM patients are diagnosed between the ages of 65–74 years [3], they often have illnesses that include components of metabolic syndrome in addition to MM. Observational studies have shown an elevated risk of developing MM among obese individuals (body mass index between 28 and 31) and among individuals with diabetes [11]. The prevalence of diabetes was between 18 and 22% at the time of MM diagnosis [12, 13]. Moreover, obesity [14] and diabetes [15–17] were reported to increase MM-related mortality. Patients treated for MM reportedly have an increased incidence of hypertension, as well as more numerous events of malignant hypertension compared to non-MM individuals. This is at least partly attributable to high-dose steroid treatment. However, even at baseline, before steroid treatment had been initiated, the prevalence of hypertension among MM patients approached 38–47% [18, 19]. Hachem et al. described a tendency toward hypocholesterolemia in MM patients, and observed that it increases with the severity of the disease [20].

The treatment of MM, including steroid therapy, chemotherapy, and bone marrow transplant, may exacerbate the features of metabolic syndrome, which is characterized by hyperglycemia, hypertension, dyslipidemia, and obesity [21]. Accordingly, recommendations have been issued for screening for metabolic syndrome among patients who underwent bone marrow transplants [22].

Much less is known about the components of metabolic syndrome in patients with SMM who are not receiving either anti-MM treatment or steroids. The question arises as to whether an increased risk of various components of metabolic syndrome in MM patients is due to the treatment or whether it could be already present at the stage of SMM due to the disease itself. The aim of this study was to examine the prevalence of diabetes mellitus, hypertension, and dyslipidemia in patients with MM and in those with SMM at the time of diagnosis (baseline), and to determine the incidences of these diseases during a follow-up of 1, 3, and 5 years in comparison to a healthy control group.

## Methods

### Patient population

We conducted a prospective historical study on three cohorts: 1) SMM patients who were monitored at Tel Aviv Sourasky Medical Center (TASMC); 2) MM patients who were treated at TASMC; 3) healthy individuals who were undergoing routine medical checkups at the Institute for Preventive Medical Examinations at TASMC and comprised the control group. The sole inclusion criterion for the three groups was age 18 years or over. Diagnoses of MM or SMM were according to the criteria of the International Myeloma Working Group [7]. Exclusion criteria were: 1) chronic treatment with steroids (except for patients with MM); 2) HIV carriage or a diagnosis of AIDS; 3) a solid tumor or another active hematological malignancy within 3 years of the initiation of the follow-up; 4) insufficient data for at least 1 year of follow-up.

### Methods

Data were collected from the medical records of patients treated in the Hematology Department and in the Institute of Special Medical Examinations at the TASMC between 2008 and 2015. Retrieved information included the patients’ age, sex, date of diagnosis of SMM or MM, date of initiation of medical treatment for MM, background diseases, medical treatment added during the follow-up (unrelated to MM), and baseline height, weight, and body mass index. Also included were the glucose, HbA1c, triglycerides, total cholesterol, high-density lipoprotein cholesterol (HDL-C), and low-density lipoprotein cholesterol (LDL-C) test results at baseline, and after 1, 3, and 5 years of follow-up.

The SMM patients were followed for at least 1 year and up to 5 years or until progression to MM, and they were monitored in the hematology clinic without receiving any medication. The SMM patients that progressed to MM were switched to the MM group in the analysis. The MM group received conventional therapy, including prednisone, at diagnosis.

The historical control group consisted of healthy individuals who were matched to the patients in the SMM and MM groups by age, sex, and length of follow-up. The same demographic, clinical, and laboratory data of the controls were retrieved from the TASMC database between 2008 and 2015.

Diabetes mellitus prior to and during the follow-up period was defined as the presence of at least one of the following: 1) an HbA1C result of 6.5% or above; 2) a record of hypoglycemic medications; 3) a diagnosis of diabetes as recorded by the treating physician. A diagnosis of hypertension prior to and during follow-up was defined according to the presence of one of the following: 1) initiation of treatment for hypertension; 2) the diagnosis of hypertension as recorded by the treating physician.

A diagnosis of dyslipidemia prior to and during the follow-up was defined as the appearance of at least one of the following: 1) an LDL-C level of 160 mg/dL or above in the absence of diabetes or hypertension; 2) an LDL-C level of 130 mg/dL or above in the presence of hypertension; 3) an LDL-C level of 100 mg/dL or above in the presence of diabetes or ischemic heart disease; 4) a triglycerides level of 150 mg/dL or above; 5) an HDL-C level lower than 40 mg/dL in males or lower than 50 mg/dL in females; 6) initiation of treatment for dyslipidemia; 7) a new diagnosis of dyslipidemia as recorded by the treating physician.

### Statistical analysis

Continuous variables with normal distributions are presented as means and standard deviations, and variables without normal distributions are given as medians and interquartile ranges. Categorical variables are presented as numbers and percentages of patients in each group. For the purpose of evaluating metabolic measures, the one-way ANOVA test was performed according to patient groups (MM, SMM, and control) by means of the Bonferroni post hoc analysis for multiple comparisons. The paired *t*-test was used to assess changes in various measures between the first and last points of follow-up.

Categorical variables were compared by the chi-squared test. Cox regression analyses adjusted for sex, age, the follow-up duration, and presence of diabetes, hypertension and dyslipidemia at baseline were performed to determine de novo incidences of diabetes, hypertension and dyslipidemia. A *P* value < 0.05 was considered statistically significant. All statistical analyses were performed using IBM SPSS Statistics 22.0 (IBM Corporation, Armonk, New York, USA.).

## Results

**Fig. 1:**
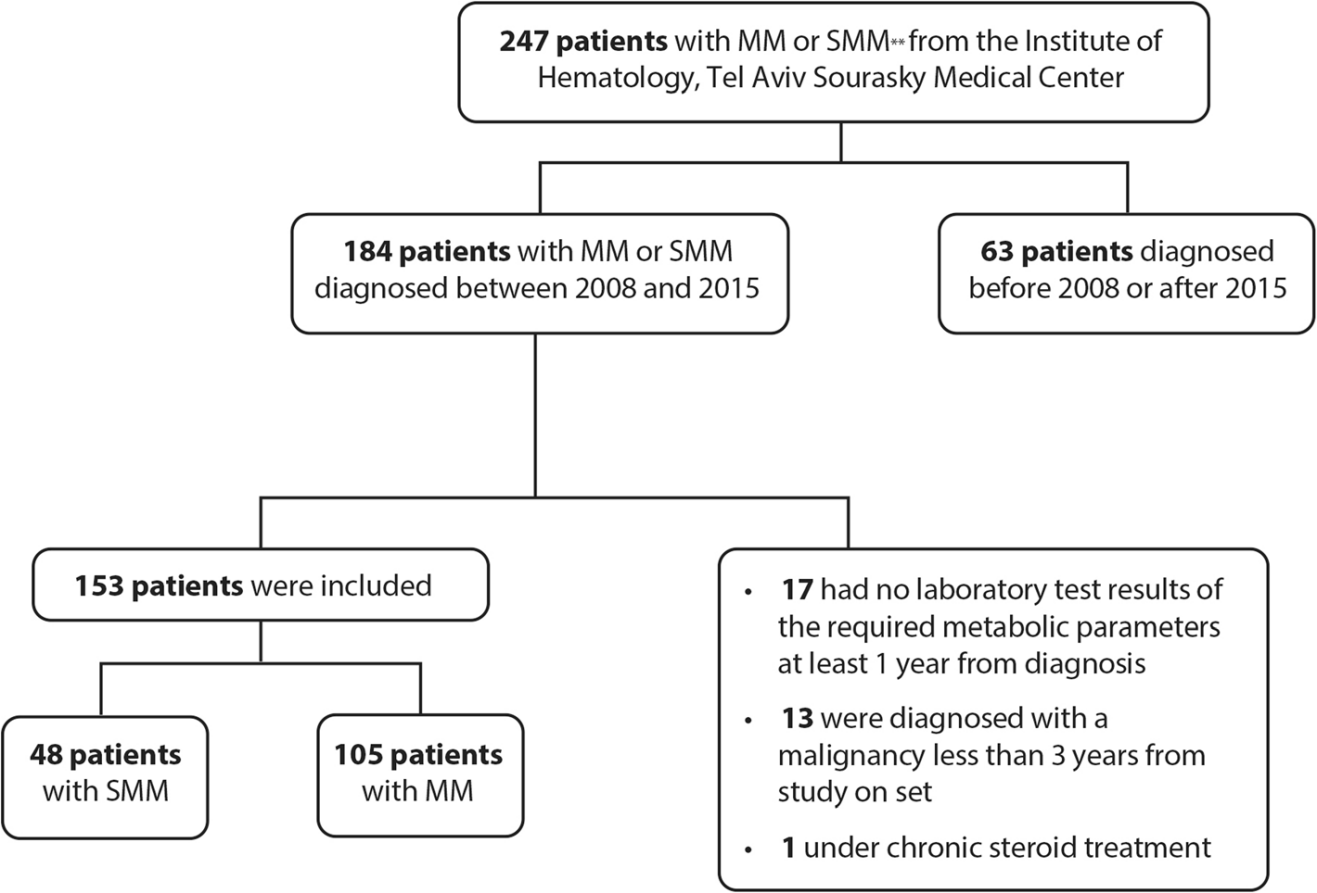
The study population. *MM* Multiple Myeloma; *SMM* Smoldering Multiple Myeloma

A total of 184 patients diagnosed with MM or SMM were treated in the hematological department of the TASMC during the study period. After excluding 31 patients who did not meet inclusion criteria (Fig. 1), the final cohort was comprised of 153 patients, 105 with MM and 48 with SMM. The mean retrospective follow-up period for the MM group was 2.85 ± 1.51 years. The mean retrospective follow-up period for the SMM group was 2.75 ± 1.68 years during which 14 out of 48 patients with SMM progressed to MM. Table 1 presents demographic and baseline clinical characteristics of the three groups. The prevalence of diabetes at diagnosis was similar in the MM and SMM groups, and significantly higher than in the control group (26.5, 25, and 11%, for the MM, SMM, and control groups, respectively, *P* < 0.001). The prevalence of hypertension and dyslipidemia were significantly higher in the SMM group compared to the control group (60.4% vs 41.3% for hypertension, *P* = 0.034, 54.2% vs 31.9% for dyslipidemia, *P* = 0.02). The prevalences of hypertension and dyslipidemia were also higher in the MM group than in the control group, but not to a level of significance (53.3% vs 41.3% for hypertension, *P* = NS, 41.3% vs 31.9% for dyslipidemia, *P* = NS). The prevalence of ischemic heart disease was 12.5, 14.6, and 6.5% in the MM, SMM, and control groups, respectively (*P* = NS).

**Table 1:** Demographic and clinical characteristics of the study population at study entry

Parameter	Multiple myeloma	Smoldering multiple myeloma	Controls	*P*-value
Number	105	48	138	
Age, yr (SD)	64.5 (12.5)	67.8 (9.4)	64.5 (4.8)	.071
Male (%)	54 (51.4)	27 (56.3)	80 (57.2)	.653
BMI, kg/m2 (SD)	27.6 (4.8)	26.2 (4.1)	27.2 (3.8)	.483
Diabetes (%)	28 (26.5)a	12 (25.0)a	11 (8.0)b	<.001
Hypertension (%)	56 (53.3)ab	29 (60.4)a	57 (41.3)b	.034
Dyslipidemia (%)	43 (41.3)ab	26 (54.2)a	44 (31.9)b	.020
IHD (%)	13 (12.5)	7 (14.6)	11 (6.5)	.153

*SD* standard deviation, *BMI* body mass index, *IHD* ischemic heart disease

^a^ and ^b^Used to discriminate within-group difference

^a^Statistically different from^b^

^ab^Intermediate group which does not differ from either ^a^ or ^b^

At baseline, the use of ACE inhibitors and hypoglycemic drugs was significantly greater in the MM and SMM groups compared to the control group (27.6, 22.9, and 8.7%, respectively, *P* < 0.001 for ACE inhibitors, and 21.9, 16.7, and 6.5%, respectively, *P* = 0.002 for hypoglycemic drugs). The use of angiotensin receptor blockers was significantly higher in the SMM group than in the control group (9.5, 22.9 and 3.6%, for the MM, SMM, and control groups, respectively, *P* < 0.001). The use of statins was significantly higher in the SMM group than in the MM and control groups (34.3, 54.2, and 28.9%, for the MM, SMM and control groups, respectively, *P* = 0.007).

Table 2 lists the laboratory test results of the three groups at baseline. The triglyceride and glucose levels were significantly higher in the MM group compared to the control group, while both the LDL-C and HDL-C levels were significantly higher in the control group than in the other two groups. There was no significant sex-related difference in metabolic parameters in the SMM and MM groups.

**Table 2:** Blood test results of the study population at study entry

Parameter	Multiple myeloma		Smoldering multiple myeloma		Controls		*P*-value
TG (mg/dL)	Mean	(95% CI)	Mean	(95% CI)	Mean	(95% CI)	
HDL-C (mg/dL)	146.0a	130.9–161.1	132.2ab	110.1–154.3	120.8b	109.5–132.1	.027
LDL-C (mg/dL)	48.4a	45.6–51.3	50.4a	45.9–54.8	57.9b	54.9–60.9	<.001
TC (mg/dL)	104.1a	96.6–111.7	95.8a	86.4–105.3	129.9b	123.5–136.3	<.001
Glucose (mg/dL)	181.2a	170.9–191.4	171.8a	160.6–183.0	212.5b	205.1–219.8	<.001
*CI* confidence interval,	108.0a	101.3–114.8	102.6ab	92.8–112.4	98.0b	95.1–100.9	.018

*CI* confidence interval, *TG* triglycerides, *HDL-C* HDL-cholesterol, *LDL-C* LDL-cholesterol, *TC* total cholesterol

^a^ and ^b^Used to discriminate within-group difference

^a^Statistically different from ^b^

^ab^Intermediate group which does not differ from ^a^ or ^b^

**Fig. 2:**
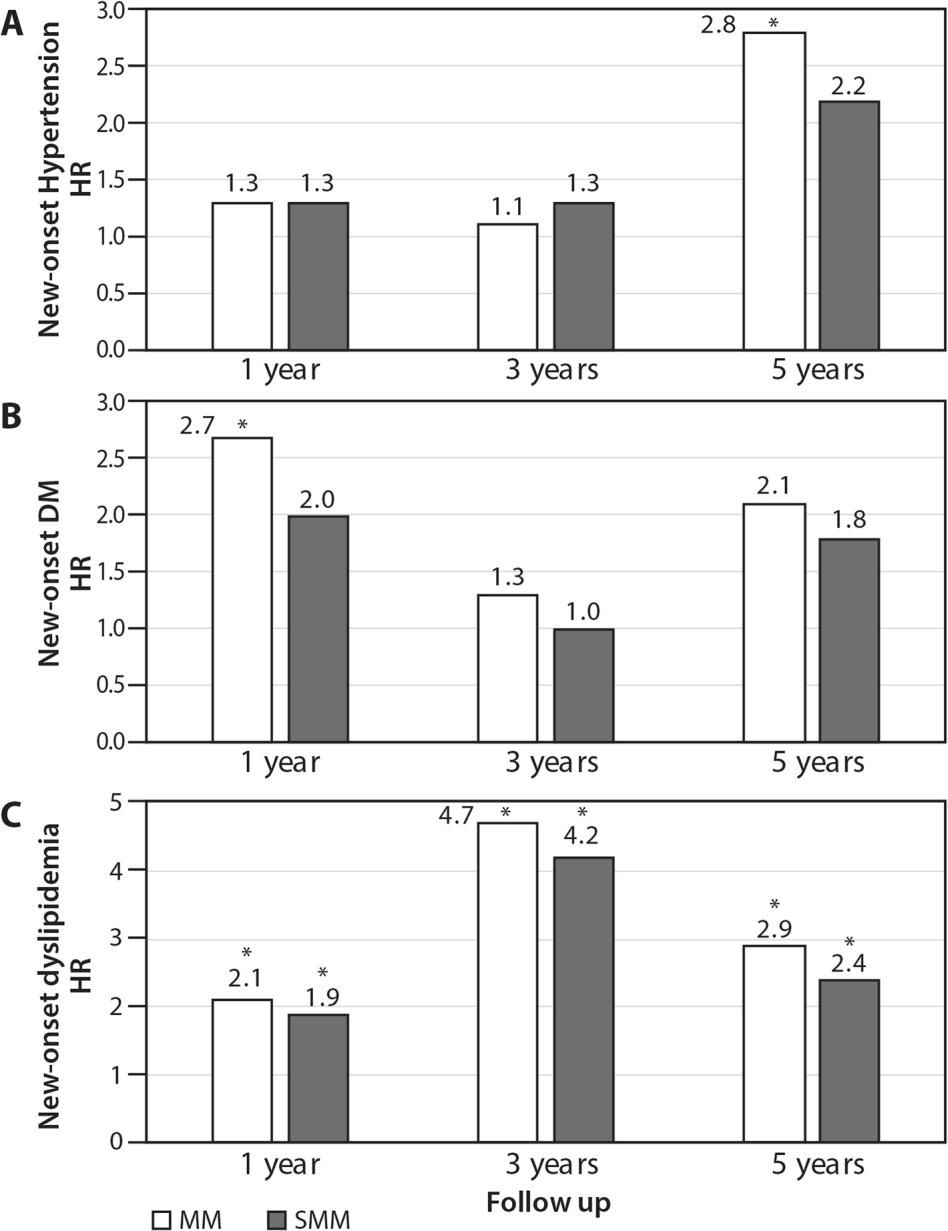
New-onset dyslipidemia, diabetes mellitus and hypertension in the multiple myeloma and in the smoldering multiple myeloma patient groups compared to the control group. *HR* hazard ratio adjusted for age, sex, follow-up duration, and the presence of dyslipidemia, diabetes, and hypertension at study entry*, MM* multiple myeloma, *SMM* smoldering multiple myeloma

A multivariate Cox regression analysis adjusted for age, sex, follow-up duration, and the presence of dyslipidemia, diabetes, and hypertension at the beginning of the study revealed that the risk of developing dyslipidemia after 1, 3, and 5 years of follow-up was greater for the MM group (Hazard ratio [HR] 2.1, 95% confidence interval [CI] 1.38–3.27; HR 4.7, 95% CI 2.32–9.61; HR 2.9, 95% 1.54–5.45, respectively) and for the SMM group (HR 1.9, 95% CI 1.14–3.14; HR 4.2, 95% CI 1.87–9.61; HR 2.4, 95% CI 1.09–5.37, respectively) compared to the control group (Table 3, Fig. 2). The risk of new-onset diabetes was greater for the MM and SMM groups than the control group, but a level of significance was reached only for the MM group (HR 2.7, 95% CI 1.17–6.13) after 1 year of follow-up (Table 3, Fig. 2). The risk of hypertension was greater for the MM and SMM groups than the control group, but, again, a level of significance was reached only for the MM group (HR 2.8, 95% CI 1.49–5.25) after 5 years (Table 3, Fig. 2).

**Table 3:** New-onset dyslipidemia, diabetes mellitus and hypertension in the multiple myeloma and in the smoldering multiple myeloma patient groups compared to the control group

New-onset disease	Study group	HR^a^ study/control	95% CI	*P*-value
Dyslipidemia				
1-year FU	MM	2.1	1.38–3.27	.001
	SMM	1.9	1.14–3.14	.013
3-year FU	MM	4.7	2.32–9.61	<.001
	SMM	4.2	1.87–9.61	.001
5-year FU	MM	2.9	1.54–5.45	.001
	SMM	2.4	1.09–5.37	.029
DM				
1-year FU	MM	2.7	1.17–6.12	.020
	SMM	2.0	0.76–5.13	.165
3-year FU	MM	1.3	0.58–2.81	.547
	SMM	1.0	0.36–2.97	.952
5-year FU	MM	2.1	0.85–5.07	.107
	SMM	1.8	0.57–5.39	.324
Hypertension				
1-year FU	MM	1.3	0.90–1.97	.151
	SMM	1.3	0.80–2.05	.306
3-year FU	MM	1.1	0.72–1.80	.575
	SMM	1.3	0.71–2.23	.432
5-year FU	MM	2.8	1.49–5.25	.001
	SMM	2.2	0.95–4.96	.068

^a^Hazard ratio adjusted for age, sex, follow-up duration, and the presence of dyslipidemia, diabetes, and hypertension at study entry

*MM* multiple myeloma, *SMM* smoldering multiple myeloma, *FU* follow-up, *DM* diabetes mellitus, *CI* confidence interval

## Discussion

This study was conducted to determine the prevalence of diabetes mellitus and hypertension among patients with SMM. The results demonstrated a higher prevalence of diabetes (25.0%), hypertension (60.0%), and dyslipidemia (54.0%) among individuals with SMM compared to a healthy control group (8.0, 41.0, and 32.0%, respectively) at baseline. After 1, 3, and 5 years of follow-up, however, individuals with sustained SMM showed an increased risk of dyslipidemia, but not of diabetes or hypertension compared to the control group. The prevalence of diabetes at baseline (26.5%) was higher for the MM patients than that for the subjects in the control group. During the follow-up, the mean glucose levels and lipid profiles deteriorated significantly among the MM patients. The prevalence of diabetes, hypertension, and dyslipidemia continued to rise during the 5-year follow-up period. After the first year of follow-up, the risk of developing diabetes was higher, as was the risk of developing dyslipidemia, the latter with a subsequent elevation after 3 and 5 years of follow-up. The risk of developing hypertension did not change until 5 years of follow-up, when it was observed to rise. Our 27.0% prevalence of diabetes at MM diagnosis is about twice the rate reported by other studies [16, 18, 23]. Our 53% prevalence of hypertension was also higher than that reported in other studies on MM patients (38.0% [18] and 47.0% [19]). There were no sex-related differences, which can be at least partially due to the relatively small groups.

Numerous studies have reported associations of diabetes with all-site cancer [24], including a large population study in Israel [25]. A meta-analysis published in 2012 showed an increased (although non-significant) risk of developing MM among individuals with diabetes [11]. Various explanations have been proposed for the association of diabetes with cancer. Among the biological factors are hyperglycemia, hyperinsulinemia, overproduction of insulin-like growth factor-1 (IGF-1), increased expression of the IGF-1 receptor, and increased secretion of inflammatory cytokines, such as interleukin 6 (IL-6) and tumor necrosis factor alpha (TNF-α) [26]. Several studies have reported associations of inflammatory markers with the incidence of type 2 diabetes [27–31]. The IGF system is clearly involved in almost every stage of development of MM, beginning with the proliferation of malignant cells and extending throughout survival, and even affecting resistance to medical treatments [32]. The inflammatory process entails part of the pathogeny of MM, and IL-6 has a particularly important role in that process [33]. Indeed, an increased level of inflammatory cytokines was associated with poor prognosis of MM [34–37]. IL-6 is considered a growth factor for myeloma cells and is also an important regulator of inflammatory proteins [37]. Moreover, several studies have shown an association between the level of IL-6 and inflammatory proteins, such as C-reactive protein and antitrypsin alpha-1, and the prognosis of MM [34–36].

Hypertension is considered as being ubiquitous among MM patients and, until recently, this was believed to be due to the older age of the patients, the fact that more men are affected with the disease, and a consequence of the treatment with steroids. However, the findings of the current study revealed that hypertension was more prevalent among patients with SMM at baseline ─ and therefore not yet treated with steroids ─ than in the control group. Inflammation may also have a role in this setting, since patients with hypertension have an increased level of inflammatory cytokines [38–40], and given the fact that the inflammatory process is an important factor in the development of hypertension [41].

According to a national health study conducted on Israelis aged 21 years and older during 2013–2015 (INHIS 3), the prevalences of diabetes, hypertension, and dyslipidemia were 8.4, 20.6, and 30.2%, respectively [42]. The data on diabetes and dyslipidemia of the current control group were similar to those values, but the prevalence of hypertension was about twice as high (41%). This may be explained by the older age of our study population (average 65 years), in which increased prevalences of these diseases are to be expected. While our MM patients had a higher prevalence and incidence of hyperlipidemia compared to the controls, similar comparisons were even seen in our patients with SMM who were in an untreated premalignant stage of MM, indicating increased cardiovascular risk in both groups.

Hyperlipidemic myeloma (HLM) is a rare variant of MM. In a review that summarized 53 cases of HLM, the typical clinical presentation was characterized by IgA myeloma, hyperlipidemia, skin xanthomas, and hyperviscosity [43]. The precise mechanism for hyperlipidemia is still controversial: according to one hypothesis, paraproteins bind to lipoproteins and, thereby, inhibit their degradation [43].

In contrast, hypocholesterolemia is fairly common in MM patients [20, 44, 45]. A case-control prospective study by Yavasoglu et al. [46] found low total cholesterol, LDL-C, and HDL-C levels in MM patients compared with controls, and noted that the lipid parameters were lower in advanced stages of disease. This suggests the hypothesis that hypocholesterolemia may be due to increased LDL clearance and utilization of cholesterol by myeloma cells.

A recent analysis from the Cancer Research Network showed a protective association between statin use and MM development [47]. This association was more pronounced after 48 months or more of statin treatment, as well as in older patients (70 years or more) regardless of statin treatment duration. Another population-based cohort study of 4957 MM patients, of whom 2294 received statin treatment, revealed a statin treatment-related 21% reduction in all-cause mortality and a 24% reduction in MM-specific mortality [48]. These findings emphasize the importance of early diagnosis and treatment of hyperlipidemia in patients with SMM and MM.

This study has a number of limitations largely due to its retrospective design. Some important data were missing, such as smoking in the present or past and details on family history of hyperlipidemia. A systematic follow-up of blood pressure measurements was not done for the MM and SMM groups, thus, individuals were considered to have hypertension only according to documentation of a diagnosis by their primary care physicians or the initiation of treatment for hypertension. In contrast, hypertension was assessed and recorded in the medical records of the individuals of the control group. Another drawback may be selection bias in the interpretation of the findings. Since the patients included in this study were being followed-up in the hematology department of a tertiary hospital, there is a greater chance that their disease would be severe and possibly with more background disease and, as a result, they may not necessarily represent the MM and SMM patients who are treated in the community. Finally, despite efforts to access all relevant clinical and laboratory data, the sample size was small, particularly at the end of the 5-year follow up. Together with missing data, this may explain the lack of statistical significance for some of the analyses.

## Conclusions

In conclusion, this retrospective cohort study showed that the MM patients had a higher prevalence of diabetes at baseline and had a higher risk to develop diabetes after 1 year, hypertension after 5 years, and dyslipidemia after 1, 3, and 5 years of follow-up compared to the non-MM controls. In addition, individuals with SMM were more prone to develop dyslipidemia after 1, 3, and 5 years of follow-up compared to the controls. These findings support the notion that characteristics of MM itself, and not only its treatment, may be associated with an increased risk of various components of metabolic syndrome and call for future prospective research of those components in SMM and MM patient populations.

## Data Availability

The data that support the findings of this study are available from database of the Tel Aviv Sourasky Medical Center, but restrictions apply to the availability of these data, which were used under license for the current study, and so are not publicly available. Data are however available from the authors upon reasonable request and with permission of the Tel Aviv Sourasky Medical Center.

## Abbreviations

MM: Multiple myeloma
SMM: Smoldering multiple myeloma
TASMC: Tel Aviv Sourasky Medical Center
HDL-C: high-density lipoprotein cholesterol
LDL-C: Low-density lipoprotein cholesterol
HR: Hazard ratio
CI: Confidence interval
IGF-1: Insulin-like growth factor-1
IL-6: Interleukin 6
TNF-α: Tumor necrosis factor alpha
HLM: Hyperlipidemic myeloma

## References

[1] Wildes TM, Campagnaro E (2017). Management of multiple myeloma in older adults: gaining ground with geriatric assessment. J Geriat Oncol.

[2] Bianchi G, Richardson PG, Anderson KC (2015). Promising therapies in multiple myeloma. Blood.

[3] Fonseca R, Abouzaid S, Bonafede M, Cai Q, Parikh K, Cosler L (2017). Trends in overall survival and costs of multiple myeloma, 2000-2014. Leukemia.

[4] Maiese EM, Evans KA, Chu B, Irwin DE (2018). Temporal trends in survival and healthcare costs in patients with multiple myeloma in the United States. Am Health Drug Benefits.

[5] Kyle RA, Therneau TM, Rajkumar SV, Offord JR, Larson DR, Plevak MF (2002). A long-term study of prognosis in monoclonal gammopathy of undetermined significance. N Engl J Med.

[6] Kyle RA, Rajkumar SV (2008). Multiple myeloma. Blood.

[7] Ravindran A, Bartley AC, Holton SJ, Gonsalves WI, Kapoor P, Siddiqui MA (2016). Prevalence, incidence and survival of smoldering multiple myeloma in the United States. Blood Cancer J.

[8] Rajkumar SV, Dimopoulos MA, Palumbo A, Blade J, Merlini G, Mateos MV (2014). International myeloma working group updated criteria for the diagnosis of multiple myeloma. Lancet Oncol.

[9] Rajkumar SV, Landgren O, Mateos M-V, Kyle R, Remstein E, Therneau T (2015). Smoldering multiple myeloma. Blood.

[10] Rajkumar SV, Larson D, Kyle RA (2011). Diagnosis of smoldering multiple myeloma. N Engl J Med.

[11] Castillo JJ, Mull N, Reagan JL, Nemr S, Mitri J (2012). Increased incidence of non-Hodgkin lymphoma, leukemia, and myeloma in patients with diabetes mellitus type 2: a meta-analysis of observational studies. Blood.

[12] Badros A, Goloubeva O, Dalal JS, Can I, Thompson J, Rapoport AP (2007). Neurotoxicity of bortezomib therapy in multiple myeloma: a single-center experience and review of the literature. Cancer.

[13] Richardson PG, Sonneveld P, Schuster MW, Stadtmauer EA, Facon T, Harousseau JL (2009). Reversibility of symptomatic peripheral neuropathy with bortezomib in the phase III APEX trial in relapsed multiple myeloma: impact of a dose-modification guideline. Br J Haematol.

[14] Calle EE, Rodriguez C, Walker-Thurmond K, Thun MJ (2003). Overweight, obesity, and mortality from cancer in a prospectively studied cohort of U.S. adults. N Engl J Med.

[15] Jin S, Wu Y, Deng S, Zhang J, Chen XQ (2014). Oxidants and antioxidants in metabolic syndrome and cancer. Oxidative Med Cell Longev.

[16] Chou Y-S, Yang C-F, Chen H-S, Yang S-H, Yu Y-B, Hong Y-C (2012). Pre-existing diabetes mellitus in patients with multiple myeloma. Eur J Haematol.

[17] Chiu BCH, Gapstur SM, Greenland P, Wang R, Dyer A (2006). Body mass index, abnormal glucose metabolism, and mortality from hematopoietic cancer. Cancer Epidemiol Biomark Prev.

[18] Chari A, Mezzi K, Zhu S, Werther W, Felici D, Lyon AR (2016). Incidence and risk of hypertension in patients newly treated for multiple myeloma: a retrospective cohort study. BMC Cancer.

[19] Song X, Cong Z, Wilson K (2016). Real-world treatment patterns, comorbidities, and disease-related complications in patients with multiple myeloma in the United States. Curr Med Res Opin.

[20] Hachem H, Favre G, Ghalim N, Puchois P, Fruchart JC, Soula G (1987). Quantitative abnormalities of lipoprotein particles in multiple myeloma. Clin Chem Lab Med.

[21] Annaloro C, Usardi P, Airaghi L, Giunta V, Forti S, Orsatti A (2008). Prevalence of metabolic syndrome in long-term survivors of hematopoietic stem cell transplantation. Bone Marrow Transplant.

[22] Defilipp Z, Duarte R, Snowden J, Majhail N, Greenfield D, Miranda J (2017). Metabolic syndrome and cardiovascular disease following hematopoietic cell transplantation: screening and preventive practice recommendations from CIBMTR and EBMT. Bone Marrow Transplant.

[23] Dimopoulos MA, Mateos MV, Richardson PG, Schlag R, Khuageva NK, Shpilberg O (2011). Risk factors for, and reversibility of, peripheral neuropathy associated with bortezomib-melphalan-prednisone in newly diagnosed patients with multiple myeloma: subanalysis of the phase 3 VISTA study. Eur J Haematol.

[24] Tsilidis KK, Kasimis JC, Lopez DS, Ntzani EE, Ioannidis JPA (2015). Type 2 diabetes and cancer: umbrella review of meta-analyses of observationlal studies. BMJ.

[25] Dankner R, Boffetta P, Balicer RD, Boker LK, Sadeh M, Berlin A (2016). Time-dependent risk of cancer after a diabetes diagnosis in a cohort of 2.3 million adults. Am J Epidemiol.

[26] Giovannucci E, Harlan DM, Archer MC, Bergenstal RM, Gapstur SM, Habel LA (2010). Diabetes and cancer: a consensus report. Diabetes Care.

[27] Duncan BB, Schmidt MI, Pankow JS, Ballantyne CM, Couper D, Vigo A (2003). Low-grade systemic inflammation and the development of type 2 diabetes. Diabetes.

[28] Pradhan AD, Manson JE, Rifai N, Buring JE, Ridker PM (2001). C-reactive protein, interleukin 6, and risk of developing type 2 diabetes mellitus. J Am Med Assoc.

[29] Schmidt MI, Duncan BB, Sharrett AR, Lindberg G, Savage PJ, Offenbacher S (1999). Markers of inflammation and prediction of diabetes mellitus in adults (atherosclerosis risk in communities study): a cohort study. Lancet.

[30] Festa A, Agostino RD, Tracy RP, Haffner SM (2002). Elevated levels of acute-phase proteins and plasminogen activator inhibitor-1 predict the development of type 2 diabetes. Diabetes.

[31] Vozarova B, Weyer C, Lindsay RS, Pratley RE, Bogardus C, Tataranni PA (2002). High white blood cell count is associated with a worsening of insulin sensitivity and predicts the development of type 2 diabetes. Diabetes.

[32] Bieghs L, Johnsen HE, Maes K, Menu E, Van Valckenborgh E, Overgaard MT (2016). The insulin-like growth factor system in multiple myeloma: diagnostic and therapeutic potential. Oncotarget.

[33] Klein B, Bataille R (1992). Cytokine network in human multiple myeloma. Hematol Oncol Clin North Am.

[34] Merlini G, Perfetti V, Gobbi PG, Quaglini S, Franciotta DM, Marinone G (1993). Acute phase proteins and prognosis in multiple myeloma. Br J Haematol.

[35] Pelliniemi TT, Irjala K, Mattila K, Pulkki K, Rajamäki A, Tienhaara A (1995). Immunoreactive interleukin-6 and acute phase proteins as prognostic factors in multiple myeloma. Finnish Leukemia Group. Blood.

[36] Alexandrakis MG, Passam FH, Ganotakis ES, Sfiridaki K, Xilouri I, Perisinakis K (2003). The clinical and prognostic significance of erythrocyte sedimentation rate (ESR), serum interleukin-6 (IL-6) and acute phase protein levels in multiple myeloma. Clin Lab Haematol.

[37] Castell JV, Gómez-Lechón MJ, David M, Andus T, Geiger T, Trullenque R (1989). Interleukin-6 is the major regulator of acute phase protein synthesis in adult human hepatocytes. FEBS Lett.

[38] Sung KC, Suh JY, Kim BS, Kang JH, Kim H, Lee MH (2003). High sensitivity C-reactive protein as an independent risk factor for essential hypertension. Am J Hypertens.

[39] Bautista LE, Vera LM, Arenas IA, Gamarra G (2005). Independent association between inflammatory markers (C-reactive protein, interleukin-6, and TNF-alpha) and essential hypertension. J Hum Hypertens.

[40] Bautista LE, López-Jaramillo P, Vera LM, Casas JP, Otero AP, Guaracao AI (2001). Is C-reactive protein an independent risk factor for essential hypertension?. J Hypertens.

[41] Solak Y, Afsar B, Vaziri ND, Aslan G, Yalcin CE, Covic A (2016). Hypertension as an autoimmune and inflammatory disease. Hypertens Res.

[42] Hayek S, Ifrah A, Enav T, Shohat T (2017). Prevalence, correlates, and time trends of multiple chronic conditions among Israeli adults: estimates from the Israeli National Health Interview Survey, 2014–2015. Prev Chronic Dis.

[43] Misselwitz B, Goede JS, Pestalozzi BC, Schanz U, Seebach JD (2010). Hyperlipidemic myeloma: review of 53 cases. Ann Hematol.

[44] Humgria VT, Latrilha MC, Rodrigues DG, Bydlowski SP, Chiattone CS, Maranhão RC (2004). Metabolism of a cholesterol-rich micromulsion (LDE) in patients with multiple myeloma and a preliminary clinical study of LDE as a drug vehicle for the treatment of the disease. Cancer Chemother Pharmacol.

[45] Kyle RA (1975). Multiple myeloma: review of 869 cases. Mayo Clin Proc.

[46] Yavasoglu I, Tombuloglu M, Kadikoylu G, Donmez A, Cagirgan S, Bolaman Z (2008). Cholesterol levels in patients with multiple myeloma. Ann Hematol.

[47] Epstein MM, Divine G, Chao CR, Wells KE, Feigelson HS, Scholes D (2017). Statin use and risk of multiple myeloma: an analysis from the Cancer research network. Int J Cancer.

[48] Sanfilippo KM, Keller J, Gage BF, Luo S, Wang TF, Moskowitz G (2016). Statins are associated with reduced mortality in multiple myeloma. J Clin Oncol.

